# Application and research progress of artificial intelligence in allergic diseases

**DOI:** 10.7150/ijms.105422

**Published:** 2025-04-09

**Authors:** Hong Tan, Xuehua Zhou, Huajie Wu, Min Wang, Han Zhou, Yue Qin, Yun Zhang, Qiuhong Li, Jianfeng Luo, Hui Su, Xin Sun

**Affiliations:** 1 Department of Pediatrics, Xijing Hospital, The Fourth Military Medical University, Xi'an, Shaanxi, China.; 2 Department of Geriatrics, Xijing Hospital, The Fourth Military Medical University, Xi'an, Shaanxi, China.

**Keywords:** Artificial intelligence, Allergic diseases, Diagnosis and prediction, management

## Abstract

Artificial intelligence (AI), as a new technology that can assist or even replace some human functions, can collect and analyse large amounts of textual, visual and auditory data through techniques such as Reinforcement Learning, Machine Learning, Deep Learning and Natural Language Processing to establish complex, non-linear relationships and construct models. These can support doctors in disease prediction, diagnosis, treatment and management, and play a significant role in clinical risk prediction, improving the accuracy of disease diagnosis, assisting in the development of new drugs, and enabling precision treatment and personalised management. In recent years, AI has been used in the prediction, diagnosis, treatment and management of allergic diseases. Allergic diseases are a type of chronic non-communicable disease that have the potential to affect a number of different systems and organs, seriously impacting people's mental health and quality of life. In this paper, we focus on asthma and summarise the application and research progress of AI in asthma, atopic dermatitis, food allergies, allergic rhinitis and urticaria, from the perspectives of disease prediction, diagnosis, treatment and management. We also briefly analyse the advantages and limitations of various intelligent assistance methods, in order to provide a reference for research teams and medical staff.

## Introduction

Allergic diseases are one of the key diseases to be researched and prevented in the 21st century, including asthma, atopic dermatitis (AD), allergic rhinitis (AR), food allergy (FA) and urticaria, etc. The prevalence of allergic diseases has increased rapidly in recent years. 40% of the population is affected. Around the world, about 300 million people suffer from asthma and 400 million from allergic rhinitis 1, 2, the prevalence of atopic dermatitis in children is about 10-20% 3, and the prevalence of food allergy is about 4-10%^4^, which places a huge psychological burden and economic pressure on humanity. Asthma is a heterogeneous disease characterised by chronic airway inflammation and airway hyperresponsiveness. Although asthma diagnosis and treatment are strictly regulated in accordance with the Global Initiative for Asthma (GINA) in various countries, the expected goals have not been achieved. Studies have shown that the overall control of asthma in Chinese children is unsatisfactory 5. Parents are not sufficiently aware of the dangers of acute asthma attacks. Among all children with asthma, only 58.7% use inhaled steroids and 71.4% use bronchodilator 6. It is clear that there is still a lot of room for improvement in the diagnosis, treatment and management of asthma. A new approach is urgently needed to change this situation.

The field of Artificial Intelligence (AI) pertains to the development of systems capable of exhibiting intelligent behaviour and the capacity to process, learn, and respond to information from data in a manner analogous to that of humans. In comparison to human intelligence, the advantage of AI is that it can integrate a large amount of data in a short period of time, automatically extract valuable information, learn the intrinsic relationship between 'input' and 'output', and transform it into appropriate models. The combination of AI and medicine has brought great convenience to human life. The application of diagnostic and monitoring models for allergic diseases can better improve the construction of the medical system, so that patients with allergic diseases can receive real-time advice on disease management, thus reducing the risk of acute attacks. At the same time, the powerful communication function of AI can realise telemedicine, bringing doctors and patients closer together, narrowing the differences caused by the disparity in medical levels around the world and effectively improving the use of medical resources. In this review, we summarize the latest research on the models developed based on AI for the prediction, diagnosis, treatment and management of allergic diseases.

## Application of AI in asthma

### Diagnosis and classification of asthma

At present, AI has been widely used in the diagnosis of asthma. The efficiency of the models developed by different researchers varies. Coughing is defined as a reflex action initiated by sensory nerves located within the respiratory tract. Collecting cough sounds in a clinical setting is difficult, some researchers have developed a smartphone app, which can collect the cough frequency of asthmatic patients during waking and sleeping hours, and believe that this app could be a new solution for objectively monitoring cough in a clinical setting 7. Similarly, Porter 8 collected cough sounds from patients (children aged 29 days to 12 years) in a real clinical environment, used a Time Delay Neural Network (TDNN) to analyse the characteristics of the cough sounds, and developed an algorithm for diagnosing respiratory diseases based on cough sound analysis. The accuracy of the algorithm was verified, and the findings demonstrated that the algorithm exhibited a high degree of concordance with paediatricians' diagnoses of asthma (Positive Percent Agreement: 97%, Negative Percent Agreement: 91%). Existing detection devices for patients' breathing sounds are complicated. For example, a peak flow meter (PFM) can only detect breathing at a certain time. Experiments have shown that AI can effectively distinguish between normal and abnormal breathing sounds in asthmatic patients, and has great potential for use by patients at home 9. A new four-channel data acquisition system was employed by researchers to collect the breath sounds of 60 individuals (30 normal subjects and 30 asthmatic patients) at four different locations on their backs. Artificial Neural Network (ANN) and Support Vector Machine (SVM) were employed for the classification of normal individuals and asthmatic patients, as well as the evaluation of the performance of each channel. The findings demonstrated that the combined channel was better than the single channel, and the highest classification accuracy (93.3%) was achieved by combining the classifier with channel 3 10.

Electronic health record (EHR) information is an integral part of an asthma patient's visit to the doctor. AI can mimic the human mind and diagnose asthma by extracting asthma-related features from EHR information. Wu^11^ developed a Machine Learning clustering model that extracted and analysed age, gender, ethnicity, body mass index, lung capacity and hormone use by mining the EHR information of 13,498 asthma patients over a period of 11 years, and ultimately identified five clinically important phenotypes that were validated to have an accuracy of 95.7% and a precision of 96.1%. In addition, Deep Learning has been shown to identify chronic cough conditions such as asthma and Upper Airway Cough Syndrome, from EHR information^12^.

Deep Learning is an important branch of Machine Learning, which can use Deep Neural Networks to simulate the decision-making and analysis ability of the human brain, automatically extracting the features of things, and the extracted deep features are more accurate and stable than those designed manually. Sang^13^ combined a wearable sensor with Deep Learning to develop a tool that can automatically identify patient's wheeze and respiration counts. The sensor was placed at nine commonly used lung auscultation sites, which can capture and denoise lung-induced vibrations in real time, and Deep Learning was applied for wheeze identification and classification, which was validated to have an accuracy of 95%, sensitivity of 96%, specificity of 93%, and the wearable sensor outperformed digital stethoscopes in both sound capture and denoising, significantly improving the efficiency of clinical physicians for wheeze recognition and monitoring. Masks are commonly used as a protective tool for patients with respiratory diseases and are the 'first' device to sense changes in breathing. Studies have shown that a smart mask with breathable, biodegradable, easy-to-fabricate, self-powered sensors as a key part of the mask can be combined with Machine Learning to differentiate between healthy patients and those with three chronic respiratory diseases (asthma, chronic bronchitis, and chronic obstructive pulmonary disease) in a population with an accuracy of 95.55%^14^. Although Deep Learning has played a role in assisting doctors in auscultating respiratory sounds, one researcher assessed the quality of 82 intelligent assisted auscultation models developed on the basis of Deep Learning using the Checklist for the Assessment of Medical AI (ChAMAI), which showed that all models ChAMAI scores were poor to moderate and identified limitations of these models in terms of heterogeneity among them and a lack of standards for data collection and interpretation of results, which may negatively affect the refinement of physicians' auscultation skills^15^. Therefore, further optimisation of Deep Learning for asthma breath sound identification and analysis is required.

Lung function testing represents a crucial diagnostic tool for asthma, offering insights into the level of asthma control and the severity of the condition. However, the interpretation of lung function test results usually depends on doctors' judgment based on expert advice, so there may be errors in judgment. To address this issue, Topalovic 16 utilized AI to evaluate the outcomes of lung function tests and compared the results with those of 120 medical professionals. The Mican Thoracic Society/European Respiratory Society guidelines were employed as the benchmark for this assessment. The results showed that, of the 6,000 reports, the doctors accurately judged only 44.6% of them, whereas all of the AI's judgments were in accordance with the gold standard. These findings demonstrate that AI can facilitate more precise assessments of lung function test results, offering a valuable adjunct to clinical practice.

The diagnosis of asthma through the use of AI is a relatively straightforward and cost-effective process. Furthermore, it has the potential to serve as a foundation for diagnosing asthma in regions where medical resources are limited. However, this method also has certain constraints. The collection of cough and breathing sounds necessitates continuous recording, which is challenging to accomplish in medical facilities with numerous patients, particularly children who are prone to non-compliance. Constructing an asthma diagnosis model based on EHR data necessitates not only a substantial amount of information storage and data analysis equipment but also the availability of highly skilled professionals who possess expertise in both information mining technology and asthma diagnosis technology to effectively process the vast array of information.

### Treatment of asthma

Glucocorticosteroids and bronchodilators are the most commonly used asthma medications, and AI can play a role in standardising the use of these two classes of medications based on a variety of patient data. Some studies have confirmed the clinical value of AI in reducing antibiotic and systemic glucocorticoid misuse in asthma patients^17^.There is a strong correlation between the breathing sounds of asthmatic patients and alterations in the airway. Consequently, some researchers employ a range of electronic or digital stethoscopes to record breathing sounds, subsequently utilising diverse analytical techniques to digitally process and transform the obtained breathing sounds into distinctive signal parameters characteristic of asthmatic patients. This enables the establishment of a correlation between the parameters and asthma. The aforementioned method is not only applicable for the diagnosis of asthma but can also, in a certain sense, contribute to the treatment of the patient by the physician. The Ic700 parameter is calculated when the frequency of the inspiratory respiratory sound is 700 Hz. Some researchers used IC700 to evaluate the therapeutic efficacy of inhaled corticosteroids (ICS) in children with asthma. The results showed that IC700 was related to the therapeutic effect of ICS 18. One researcher 19 developed a model to explore the relationship between the therapeutic response of asthma patients to oral or intravenous glucocorticoids and asthma phenotypes, and the results showed that factors such as age, height, asthma phenotype, lung function level and baseline eosinophil level correlated with patients' clinical response to glucocorticoids. The development of this model provides clinicians with a theoretical basis for deciding whether or not to use systemic glucocorticosteroids, thereby mitigating the potential negative effects of long-term systemic glucocorticosteroid use in patients.

Allergen Immunotherapy (AIT) is a key modality in the treatment of respiratory allergic diseases, but still has the limitations of long treatment time, high cost and variable efficacy from person to person, so poor efficacy can be minimised or avoided if efficacy is predicted using available patient data prior to AIT treatment. One researcher proposed an efficacy prediction model for AIT based on Machine Learning. Clinical data from 390 asthmatic patients (with or without rhinitis) aged 4-17 years treated with AIT (subcutaneous specific immunotherapy) were collected and analysed with the prediction model to test the validity of the model, and the results showed that the accuracy of the model in predicting the efficacy of AIT was 87. 18%, the sensitivity was 93.55% and basophil count, sIgE/tIgE (Der p) and sIgE/tIgE (Der f) were also found to play a key role in predicting efficacy^20^.

There is no consensus on how to determine the duration of asthma biologics. Inselman^21^ used Elastic Network Logistic regression, Random forests and Gradient boosting machines to predict the risk of asthma worsening after stopping asthma biologics, with the primary outcome of successful discontinuation being the absence of asthma worsening within 6 months of stopping medication. The study was based on basic information on 3057 asthma patients, including age, sex, race/ethnicity, place of residence, insurance type, household income, as well as the number of visits to the doctor in the 6 months before stopping the medication, the duration of use of biologics, the season of stopping the biologics, and the presence of any comorbidities. The predictive efficiency of the models was assessed using indicators of area under the curve (AUC), sensitivity and specificity, and the results showed that the Gradient boosting machine model had the highest AUC value (0.74) in predicting the risk of asthma worsening after discontinuation of biologics.

Although such methods can help doctors determine in advance whether a certain drug is suitable for a patient's treatment, achieve precise treatment, and reduce the patient's psychological burden and financial pressure, these algorithmic models are based on a single physiological indicator of the patient and give treatment recommendations with reference to the patient's physiological state or indicators at a certain moment or stage. They do not transcend time and space, so such methods need further improvement.

### Forecasting and management of asthma

Asthma is a chronic disease, characterised by long-term, non-communicable and difficult-to-cure characteristics. The management of asthma necessitates not only a high degree of patient compliance and literacy but also regular monitoring of symptom remission by respiratory specialists. In addition, when necessary, patients should receive general knowledge education. To achieve precise long-term management, further development is essential. With this in mind, many scientists have developed intelligent tools to predict and manage asthma.

Machine learning has been identified as having considerable potential in the management of allergic diseases, such as asthma. A study was conducted to ascertain the differences between three common Machine Learning algorithms: Naive Bayes algorithm, Bayesian Network and SVM, in predicting acute attacks in asthmatic patients. The results demonstrated that the accuracy of the three models was 0.77, 1.00, and 0.80, respectively^22^. Li^23^ developed a classifier based on Machine Learning to predict asthma, which was shown to have an accuracy of 72.5%, a figure significantly higher than that of five common prediction models, including SVM(69.80%) and SVR(64.01%). Furthermore, Machine Learning models have the capacity to predict acute asthma attacks based on information from an asthma patient's EHR^24^. Nevertheless, the application of Machine Learning in the management of asthma remains challenging. Anne^25^ et al. compared the performance of models developed based on Machine Learning and Logistic regression models in the context of home management of asthmatics. Their findings revealed that the AUC of the best-performing XGBoost, a gradient boosting-based Machine Learning algorithm, was 0.58, while that of the Logistic regression model was 0.88, and the researchers concluded that Machine Learning models tend to generate more extreme risk predictions. Patients with severe asthma frequently require hospitalisation, which places an additional financial burden on the patient. It is therefore imperative that medical practitioners intervene at the earliest possible stage to prevent the condition from worsening. Lopez^26^ deployed four Machine Learning algorithms and one Deep Learning model to forecast the probability of hospital readmission of asthma patients. The performance of these five models was then evaluated through a comparative analysis. The experimental subjects comprised 1,893 asthma patients and 3,901 chronic obstructive pulmonary disease (COPD) patients. The findings revealed that the Multilayer Perceptron, a Deep Learning method, exhibited optimal sensitivity and specificity in predicting hospitalisation in patients with asthma and COPD. It is evident that asthma management tools developed based on Machine Learning require validation with a greater number of samples.

It is evident that a range of environmental factors have the capacity to exacerbate asthma symptoms. Such factors include, but are not limited to, dust, smoke, weather conditions, and pollen. A significant number of research teams have utilised various AI techniques in conjunction with environmental survey instruments to collect environmental conditions in which asthma patients reside. These teams have employed predictive analytics to forecast events such as acute asthma exacerbations, Emergency treatment, and hospitalisations. In their study, Hyemin^27^ employed a prediction model, developed using Recurrent Neural Network (RNN) and Long Short-Term Memory (LSTM) algorithms, to forecast the number of emergency and outpatient asthma patients. This model was trained on data relating to air pollutants, weather changes, and pollen concentration, and utilised a quantitative approach to assess the significance of environmental factors in asthma attacks. The results show that LSTM performs best in terms of prediction performance, with R2 of 0.650 and 0.723 for predicting the number of emergency and outpatient asthma patients, respectively. The development and utilisation of the model have the potential to serve as an early warning system for individuals with asthma. This system aims to empower individuals with an intuitive understanding of the risk of their own acute asthma attacks based on environmental information, thereby reducing the frequency of hospital visits.

Conversely, the model's capacity to predict the number of asthma patients attending emergency and outpatient clinics on a daily basis has the potential to optimise hospital management. This prediction can inform the strategic allocation of resources, such as the number of attending doctors, in anticipation of adverse weather conditions. This, in turn, can mitigate the adverse impact of imbalanced patient-physician ratios and enhance the patient experience.

An intelligent assistant for the daily management of asthma can monitor the patient's condition and lung function by collecting various data, including the patient's general symptoms and lung function data. It can provide personalised treatment recommendations in real time and early warnings of worsening conditions or acute attacks. The asthma management model, which is constructed by combining mobile devices with Machine Learning algorithms, is not only simple to operate but also requires equipment that is readily available.

## Application of AI in atopic dermatitis

Atopic dermatitis (AD) is a chronic, recurrent, inflammatory skin disease characterised by dry skin, chronic eczema-like damage and pronounced itching 28. The itching associated with AD can result in poor sleep quality, which in turn can lead to the development of anxiety, depression, suicidal ideation, and other psychological disorders. This is based on the concept of the "brain-skin " axis 29, 30, Some studies have even found that the recurrence rate of AD can reach 75.9% 31, which seriously affects people's physical and mental health. From the perspective of atopic march, AD is considered the "initial stage" of allergic diseases such as allergic rhinitis and asthma. Some children with AD will see their symptoms disappear. However, a 9-year follow-up study found that approximately half of children with AD will develop asthma, and approximately two-thirds of them will be at risk of developing allergic rhinitis in the future 32. The aetiology of AD remains uncertain, although numerous factors have been identified as potential contributors to its onset. It is very meaningful to intervene in these influencing factors before AD begins to prevent the onset and development of AD. As early as 1999, researchers used Neural Network technology to develop a predictive model for AD 33. Dong 34 utilised the S-PRESTO study cohort and Machine Learning techniques to investigate the risk factors and potential mechanisms associated with mothers of children with AD during the pre-pregnancy stage. The findings indicated that maternal alcohol consumption prior to pregnancy and the presence of depressive symptoms during pregnancy were associated with an increased risk of eczema and rhinitis, the model suggests that higher maternal blood neopterin and higher child blood dimethylglycine may serve as protective factors against the onset of early childhood wheezing. In addition, Machine Learning can also predict the occurrence of AD through human transcriptome and intestinal microbial data 35.

The researchers employed artificial intelligence (AI) technology not only to predict the occurrence of AD, but also to assess its severity. A research team utilised Machine Learning in order to conduct an in-depth phenotypic analysis and to identify 130 factors that are associated with the severity of AD in adolescents and adults. The findings indicated that gender, age, exercise, and active or passive smoking were linked to the severity of AD in patients aged 12-89 years 36. Patella 37 employed an Artificial Neural Network to examine the interrelationship between meteorological shifts, environmental contaminants, and the severity of AD. The researchers discovered a positive correlation between outdoor temperature and precipitation and AD severity. Furthermore, the model demonstrated an accuracy rate of 75.46% in predicting AD severity.

AD can manifest in a multitude of ways, and medical practitioners typically arrive at a diagnosis through an analysis of the patient's medical history, family history, and physical examination. Currently, the commonly used diagnostic criteria mainly include the Hanifin-Rajka criteria 38, the Williams criteria 39 and the clinical diagnostic criteria for AD in Chinese children proposed by Yao Zhirong 40, 41. Despite the existing diagnostic criteria being relatively mature, the clinical manifestations of some patients are atypical, which can readily result in misdiagnosis. Huang 42 were the first to establish an association between AI and hyperspectral imaging (HSI), which has the potential to identify a range of diseases, including AD, psoriasis and skin cancer. The K-fold cross-validation value was 7, the sensitivity was 90.72%, the specificity was 96.76%, and the F1 score was 90.08%. In comparison to conventional detection techniques, this approach offers the benefits of efficiency and non-invasiveness. Multiphoton tomography (MPT) is widely used in the diagnosis of skin diseases, However, the analysis of MPT data is a time-consuming process that requires the expertise of trained professionals. Consequently, some researchers have linked MPT with convolutional neural networks (CNNs) and correctly identified 97% of AD among 3663 images of living cells (including AD images and images of skin from healthy individuals 43. The severity of the disease is the most important basis for the determination of the treatment plan, which is of great help in the realisation of precision medicine. Commonly used methods for evaluating the severity of AD include the Scoring of Atopic Dermatitis (SCORAD), the Eczema area and severity index (EASI), and the Visual analogue scale (VAS), but these methods have disadvantages such as being incomplete and cumbersome to operate. As a result, many researchers have developed algorithmic models for AD diagnosis and severity differentiation using Artificial Neural Networks, Convolutional Neural Networks and other technologies. Maulana 44 compared the ability of five Deep Learning models to differentiate the severity of AD and found that the ResNet50 model had the highest accuracy (89.80%). Bang 45 used a Convolutional Neural Network (CNN) to develop an algorithm that can identify erythema, papules, epidermal peeling and lichenification on the skin of AD patients. The results of this algorithm were compared with those of five dermatologists, and it was found that the algorithm had high accuracy, sensitivity and specificity. A recent study using an Unsupervised Clustering algorithm confirmed the important role of AD severity in distinguishing between AD types (idiopathic and vascular) 46.

Currently, the treatment of AD is mainly a 'step therapy' approach based on the severity of the disease (mild, moderate and severe). Biological agents have a good therapeutic effect in patients with moderate to severe AD, are well tolerated and have a low incidence of adverse events 47. They have been approved for marketing in China in 2020. However, biological agents have the disadvantage of long treatment cycles and high prices. Once a drug is needed, it must be continued, and the therapeutic effect varies from person to person. It is therefore necessary to test the patient's response before starting treatment. Jashin 48 used four Machine Learning methods to predict factors influencing the efficacy of dupilumab in 419 AD patients using dupilumab. The results showed that the most common factors for non-response to dupilumab were the use of ibuprofen and AD patients with a Charlson Comorbidity Index of 3-4. However, this model cannot analyse non-medical factors (such as cost and access) for discontinuation of dupilumab. Machine Learning also has a role to play in screening and identifying new drugs for AD. Wang 49 found from databases that caffeoylmalic acid (CMA) can inhibit key therapeutic targets of AD (TNF-α and IL-4). Using convolutional neural networks, molecular simulations and conformational alignment, they found that CMA can change the protein conformation by binding to TNF-α and IL-4, thus determining that CMA is an inhibitor of TNF-α and IL-4. The results show that CMA is a potential dual TNF-α and IL-4 inhibitor for the treatment of AD. In the future, more and more researchers will use AI to find new treatments for AD.

AD is a chronic and relapsing disease that requires total management for optimal effectiveness. The management process mainly involves doctors helping patients to identify the causes of the disease, adjusting treatment plans, explaining precautions and side effects, and educating patients. Researchers used a high-resolution wrist activity recorder in conjunction with a recurrent neural network to record the number of nighttime scratches in AD patients. To begin with, the high-resolution wrist activity recorder identifies the wrist state, which is recorded as no activity, one-handed activity, or two-handed activity. Then, a recurrent neural network classifier is used to distinguish wrist movements from scratching and other movements, in order to quantify the number of scratches 50. Good patient education can improve patient compliance and quality of life, and reduce doctor-patient conflict. ChatGPT is a natural language processing model that can continuously and qualitatively answer patients' medical questions. Sulejmani 51 collected 99 questions (including causes, prognosis and treatment of AD) commonly asked by AD patients. The answers provided by ChatGPT and professional physicians were scored using a Likert scale. The results showed that the overall quality and reliability of ChatGPT responses were higher than those of professional physicians, and that ChatGPT can play a role in the management of AD patients. Mobile health is also playing a role in the management of people with AD. One study compared scores on the Patient Oriented Eczema Measure (POEM) before and after AD patients used a mobile health app to demonstrate the feasibility and potential impact of the app. The results showed that after AD patients used the mobile health app for a period of time, their POEM scores decreased significantly (P<0.001) and the severity of the disease decreased significantly 52.

AI has applications in exploring the influencing factors of AD, identifying skin symptoms, screening treatment targets, and recording the number of scratching times. These models have the advantages of high accuracy and short time consumption, and are suitable for use in areas with underdeveloped medical resources. However, there are limitations to these methods. For example, the model used to identify skin symptoms has certain limitations when applied to black people. Therefore, these models still need to be tested on the population to provide real-world data to increase people's trust in AI.

## Application of AI in food allergies

A food allergy (FA) is defined as an immune response that is harmful to the body following exposure to a specific food. The allergic response can be mediated by either IgE, non-IgE, or a combination of both mechanisms 53, 54. The most common manifestations of FA are changes in the skin (such as urticaria and diffuse itching), digestive system (such as nausea and vomiting), upper respiratory tract (such as sneezing, rhinorrhea, and congestion), and lower respiratory tract (such as dyspnea and chest tightness). In cases of severe illness, there may be circulatory changes, such as hypotension and syncope, which can be life-threatening. In response to this, some scientists have utilised AI to develop an algorithmic model for predicting the occurrence of FA. The existing research evidence substantiates the assertion that the intestinal microecology in early life is associated with the development and progression of FA 55, In light of this understanding, Ahmed 56 conducted a comparative analysis of the LSTM network with several prevalent Machine Learning models (Hidden Markov Model, Multi-Layer Perceptron Neural Network, Support Vector Machine, Random Forest, and LASSO regression) in order to ascertain their capacity to predict FA based on the longitudinal intestinal microecological data of children aged 0-3 years. The findings indicated that the LSTM model exhibited the most optimal predictive performance. Dimitrov 57 developed a server called AllerTOP using the k-nearest neighbour algorithm (KNN), which can predict allergens and allergen exposure routes based on the main chemical properties of amino acid sequences with a sensitivity of up to 94%. Furthermore, some researchers have employed EHR and Machine Learning to develop a predictive model for FA. The researchers identified two significant risk factors for the development of FA in children: the systemic use of antibiotics during pregnancy or infancy (OR: 1.93) and a previous diagnosis of atopic dermatitis (OR: 2.86) 58. These factors contribute to a reduction in the incidence of FA.

The diagnosis of FA is typically based on a comprehensive medical history and the results of laboratory tests, including SPT, total IgE, and allergen sIgE. An oral food challenge (OFC) represents the gold standard for diagnosing FA. Nevertheless, the procedure carries certain risks for children, and statistical evidence indicates that only 10% of countries rely on this method to diagnose FA 59. Kuniyoshi 60 demployed three Machine Learning algorithms (linear regression, linear support vector machine, and gradient boosting) to predict the OFC results of orally heated eggs for the first time, utilising common laboratory test results as input. Following several training sessions, the area under the curve for both the linear regression and linear support vector machine algorithms exceeded 0.8. Additionally, Machine Learning models have been utilised for the purpose of predicting the severity of OFC. The findings of the research indicate that basophil activation represents the most significant predictor of OFC severity 61. Furthermore, Lucy 62 put forth a Natural Language Processing (NLP) technique that is capable of identifying children with FA. Utilising NLP, the researchers identified FA-related terminology within EHR from primary care providers. This included allergies, eczema, sibling history and age, as well as foods associated with allergies, such as eggs and peanuts. Despite the development of diagnostic models for FA by the aforementioned researchers, their efficacy remains to be seen in large-scale trials. Furthermore, the promotion of these models will require a significant investment of time to overcome the public's skepticism regarding AI.

AI also plays a role in the treatment of patients with FA. Existing studies have shown that AI can already propose suitable dietary plans to patients in combination with health history 63. In the context of wheat allergies, AI has the capacity to detect even the most minute traces of wheat present in foodstuffs. As an illustration, Fourier transform infrared spectroscopy (FTIR) in combination with Machine Learning can detect minute traces of wheat that have been mixed with corn flour 64; Furthermore, a smartphone equipped with a convolutional neural network has been shown to achieve an accuracy rate of 99.1% in the detection of minute traces of wheat in food 65. The development and utilisation of these instruments has constituted a significant benefit for individuals with wheat allergies. In the context of therapeutic target screening, Hei 66 used PandaOmics, an AI-driven target discovery platform, to identify common therapeutic targets for FA, asthma and eczema. These included IL4R, IL5, JAK1, JAK2, JAK3 and NR3C1. Some drugs targeting specific targets have been approved for the treatment of asthma and eczema, and RNF19B is regarded as a promising new target for treatment.

The application of AI in the field of FA can be broadly classified into two main areas. On the one hand, AI is utilised to forecast the likelihood of developing FA, the results of OFC, and to identify FA patients based on the patient's existing data (such as intestinal microecology, EHR, laboratory test results, etc.). Conversely, AI is employed to identify the specific food allergens present in a patient's diet and to devise an appropriate dietary plan tailored to their specific needs. Furthermore, AI is involved in the exploration of potential treatment targets for FA. Nevertheless, these techniques are encumbered by significant limitations, including high costs and the potential for data breaches, particularly in the context of intestinal microecological testing. Consequently, they have not yet been widely adopted in clinical settings.

## Application of AI in allergic rhinitis

Patients with allergic rhinitis (AR) frequently present with symptoms such as nasal congestion, rhinorrhea, sneezing, and nasal itching at regular times of the year, which markedly reduces their quality of life. Air pollution represents a significant trigger for AR. Consequently, numerous researchers have employed specific air pollution indicators to anticipate the onset of AR. Huang 67 used a Random Forest model to predict the probability of children developing AR in adolescence based on the mother's air pollution situation before giving birth (within one year before birth). This was done as part of a 14-year follow-up of 1,439 mother-child pairs. The findings indicated that prenatal exposure to NO₂ and fluctuations in its concentration over time play a pivotal role in the pathogenesis of AR prior to adolescence. The area under the curve (AUC) for the model's prediction of AR was 0.84, which is of great significance in reducing the occurrence of AR. Jeon 68 employed a spatio-temporal graph Convolutional Neural Network (MST-GCN) to examine the correlation between daily PM10 concentrations and AR, AA and AD. This approach enables the estimation of the daily number of patients with allergic diseases such as AR, which offers valuable insight for hospital management.

Furthermore, AI plays an invaluable auxiliary role in the diagnosis of AR. Dai 69 applied five common Machine Learning methods to diagnose AR based on the patient's medical history, clinical symptoms, laboratory test results, and imaging test results, and evaluated the sensitivity, specificity, and accuracy of each method. The results demonstrated that the ensemble learning algorithms ARF-OOBEE and GC Forest exhibited superior overall evaluation scores in comparison to the other algorithms. Moreover, some researchers have developed a clinical decision support system (CDSS) to assist primary clinicians in diagnosing AR, with an accuracy rate of 88.31% 70. It is not uncommon for patients with allergic rhinitis (AR) to experience a recurrence of symptoms that ultimately result in the formation of nasal polyps. Betul 71 developed a Deep Learning model for identifying nasal polyps, in which the Convolutional Neural Network (CNN) with the best performance demonstrated an accuracy rate of 98.3%. Nasal cytology is a valuable tool for distinguishing between rhinitis subtypes, with an accuracy rate of 69.5% in diagnosing nasal diseases 72, However, the analysis of results is a time-consuming process. Consequently, some researchers have integrated nasal cytology (NC) with Machine Learning in order to differentiate between the various subtypes of AR. This approach has the potential to enhance diagnostic precision and inform the development of targeted therapeutic strategies 73. Furthermore, AI is involved in the identification of potential AR therapeutic targets. Byun 74 employed an AI-based drug discovery platform to identify potential pharmaceutical agents that inhibit the suppressor of cytokine signalling 3 (SOCS3) for the treatment of AR. They utilised an AI-based drug discovery platform to screen 20 Hit compounds that bind to the SH2 domain of SOCS3. The compounds were subsequently validated in an AR mouse model, wherein it was observed that Hit compound 5.8 exhibited the capacity to mitigate nasal symptoms and diminish the levels of inflammatory cytokines in the murine subjects.

AR is a chronic and recurrent disease that requires regular medication when symptoms occur. Lisha 75 used a smartwatch to monitor patients' daily medication intake and nasal symptoms. The patients were randomly assigned to either the intervention group or the control group. Both groups were required to take antihistamines on a daily basis. The intervention group employed the use of a smartwatch for the administration of their medication. In the event that the smartwatch failed to detect the patient's medication regimen, it would transmit the relevant data to the physician, who would then issue a reminder to the patient in the event that the patient had not taken the medication for a period exceeding two days. Furthermore, the smartwatch will also provide a score for the patient's daily symptoms. Following a one-month period, a comparison was made between the frequency of medication and the severity of symptoms experienced by the two groups of patients. The findings revealed that the number of oral antihistamines consumed by patients in the intervention group was markedly higher than that of the control group. Additionally, the nasal symptoms of patients in the intervention group were significantly more pronounced than those of the control group. The results of this experiment demonstrate the potential of the smartwatch in the management of AR patients. South Korea investigated the number of adolescent patients with AR who had suicidal and desperate thoughts, and found that compared with non-AR patients, AR patients were more likely to have suicidal and desperate thoughts 76. In light of the aforementioned circumstances, Hojae 77 used a Random Forest model to ascertain the suicide risk of AR patients within the age range of 13-18 years. The researchers identified depression, stress, academic performance, age and alcohol consumption as the primary factors associated with suicide among AR patients. The model demonstrated an 83.33% sensitivity to predicting suicide risk among 833 AR patients. The development of this model has the potential to reduce suicidal behaviour in AR patients and provide a theoretical foundation for the formulation of suicide prevention strategies.

The application of AI in AR is primarily evident in its utilisation for predictive, diagnostic and managerial purposes. The advancement of these models has the potential to facilitate targeted intervention prior to the onset of AR, thereby reducing the number of AR patients and alleviating the burden on medical care. Conversely, intelligent management tools can assist medical practitioners in the management of AR patients, thereby improving patient medication compliance and alleviating symptoms in a more effective manner. This approach can also improve adolescents' mental health and reduce patients' psychological pressure and economic burden. Nevertheless, these models also present certain challenges, including high costs and the potential for privacy breaches, as evidenced by the use of smartwatches.

## Application of AI in urticaria

Urticaria is a prevalent dermatological condition associated with the immune system, characterised by the formation of wheals and pruritus. The condition is typically categorised according to its aetiology and clinical course, with the following classifications being employed: acute spontaneous urticaria, chronic spontaneous urticaria and induced urticaria 78. During the initial consultation of patients with chronic spontaneous urticaria, the doctor should score the severity and activity of the disease. The most commonly employed scoring methods include the Urticaria Activity Score 7 (UAS7), which assesses the number of wheals and the degree of itching in the patient within one week. The number of wheals is determined by the physician's observation of the patient's skin symptoms and is classified into four categories: none (0/24h), mild (<20/24h), moderate (20-50/24h), and severe (>50/24h). This method has limitations such as being time-consuming and unstable, and doctors may encounter challenges in accurately counting hives and quantifying lesion area. Therefore, Carthy 79 developed an image processing model called Legit.Health-UAS-HiveNet using a convolutional neural network, which can help doctors fill in the UAS score more quickly and accurately, and the model also has the ability to perform preliminary analysis in patients with chronic spontaneous urticaria with dark skin. Skin Prick Test (SPT) is one of the methods used to detect allergens and plays a role in the diagnosis of IgE-mediated allergic diseases such as asthma, atopic dermatitis, allergic rhinitis and urticaria. In interpreting the results of SPT, the physician must utilize a ruler to quantify the diameter of the wheal, thereby identifying the specific allergen responsible. However, this approach inherently introduces a degree of measurement error, which can potentially impact the accuracy of the diagnosis. In order to enhance the precision of measurement results and reduce the time required for interpretation, Lee 80 employed image processing technology and a Deep Learning model to segment lesion images (including wheals and erythema) uploaded from smartphones. The results demonstrated that the model exhibited a sensitivity of 56.21% and a specificity of 99.95% for wheal measurement, and a sensitivity of 57.87% and a specificity of 97.97% for erythema measurement. Clustering analysis represents a specific instance of unsupervised learning within the broader domain of Machine Learning. The method allows for the division of the totality of objects into disparate clusters or groups, according to specific features. Thus, objects within the same group exhibit similarities in their features, whereas those in disparate groups display marked differences. Consequently, this approach is frequently employed for the purpose of diagnosing or differentiating between subtypes of a disease 81. The application of cluster analysis by Murat^82^ enabled the successful distinction of four subtypes among 431 urticaria patients. This finding underscores the pivotal role of cluster analysis in the identification of disease subtypes.

The application of AI in urticaria is mainly reflected in the estimation of the severity of the condition. The development of these methods can help doctors make more accurate judgments about the condition of urticaria patients during clinical diagnosis and treatment, and achieve personalised treatment.

At the same time, the application of auxiliary tools also shortens the time patients spend and reduces their financial burden. However, these auxiliary tools may also have certain limitations in practical application. For example, they may place high requirements on the image acquisition equipment of patients or doctors, requiring photographic equipment with high definition and powerful image storage capabilities. This is an additional expense for hospitals or patients, so further optimisation of these models is required in the future.

## Figures and Tables

**Table 1:** Effects of artificial intelligence for the application of asthma.

Year	Author	Specific Artificial Intelligence	Reference	Diagnose/prediction/treatment/management	Efficiency
1999	Rietveld, et al	ANN	83	Diagnose	As much as 95% of the training vectors could be classified correctly, and 43% of the test vectors.
2010	Wendy, et al	Cluster analysis	84	Diagnose; somatotype	Five groups were identified.
2014	Mattia, et al	LR; RF; ABN	85	Prediction	AUROC: 0.76-0.82
2017	Anirban, et al	RF	86	Diagnose; somatotype	SE: 80%; SP: 75%
2017	Joseph, et al	NBC; ABN; SVM	22	Management	SE: NBC (0.8); ABN (1.0); SVM (0.84) SP: NBC (0.77); ABN (1.0); SVM (0.80) AC: NBC (0.77); ABN (1.0); SVM (0.80)
2018	Harsheen, et al	NLP	87	Prediction	SE:86%; SP:98%; PPV:88%; NPV: 98%.
2018	Fontanella, et al	JDINAC	88	Diagnose	SE:84%; SP:87%
2018	Md Ariful,et al	ANN; SVM	10	Diagnose	AC: 2-channel (89.2±3.87%); 3-channel (93.3±3.10%)
2018	Huffaker MF, et al	RF	89	Management	SE:47.2%; SP:96.3%; AC:87.4%
2018	Oletic, et al	hidden Markov model	90	Management	SE:89.34%; SP:96.28%; AC:94.91%
2019	Porter, et al	TDNN	8	Diagnose	PPA:97%; NPA:91%
2019	Ghulam, et al	SVM; KNN	91	Diagnose	SVM: SE (73±11%); SP (74±12%); PPV (75±8%) KNN: SE (72±13%); SP (72±14%); PPV (73±10%)
2019	Dimitris, et al	RF	92	Diagnose	Precision:97.7%
2019	Katsuyuki, et al	DNN; SVM	93	Diagnose	AC (When based on symptom-physical signs): DNN (0.68); SVM (0.60) AC (When based on symptom-physical signs, biochemical findings, lung function tests, and the bronchial challenge test): DNN (0.98); SVM (0.82)
2019	Wei, et al	SVM	94	treatment	SE:62%; SP:87%; AC:81%
2019	Roghaye, et al	Supervised learning	95	Management	AC: 91.66%
2019	Shaoxia, et al	mobile application	96	Management	Children in the experimental group had better improved adherence, decreased respiratory tract infections, days of antibiotic use, days of school absence, parental work loss, and medical expenses.
2021	Saurav, et al	XGBoost	97	Prediction	ANSA: 0.43
2021	Balamurali, et al	BiLSTM	98	Diagnose	AUROC: when classifying coughs (0.84); when classifying subject for respiratory pathology (0.91)
2021	Honorata, et al	RCNN	9	Diagnose	AUROC: Continuous Phenomena Index (between AA and AN, 0.94); All Phenomena Index (between NN and AN, 0.91)
2022	Moslemi, et al	SVM	99	Diagnose	AC: In a model including all CT features (80%); In the model with only CT airway features (81%). F1-Score: In a model including all CT features (66%); In the model with only CT airway features (68%).
2023	Shim, et al	artificial intelligence (AI)-based cough count system	7	Diagnose; Prediction	The strong correlation between cough counts using the AI-based algorithm and human experts, and other indicators of patient health status.
2024	Kang, et al	RF	100	Diagnose	AUROC: conventional model (0.856); random forest (0.950)
2024	Jiang, et al	SVM	101	Diagnose	STK11IP may serve as a diagnostic marker for individuals with the two conditions.

**Table 2:** Effects of artificial intelligence for the application of atopic dermatitis.

Year	Author	Specific Artificial Intelligence	Reference	Diagnose/prediction/treatment/management	Efficiency
1999	Takahashi, et al	NNA; MLA	33	Prediction	NNA: AC:96.4%; SE:88.6%; SP:99.5% MLA: AC:82.3%; SE:75.1%; SP:82.6%
2018	Moreau, et al	Recurrent Neural Networks	50	Management	F1-score:68%
2020	Patella1, et al	ANN	37	Prediction	AC:75.46%
2021	Park, et al	CNN; RF; SVM	102	Diagnosis	AC: NN (97%); RF (92%); SVM (86%)
2021	Maintz, et al	Machine learning-gradient boosting	36	Prediction	AUROC:0.71[95% CI, 0.69-0.72]
2021	Guimares, et al	CNN	43	Diagnosis	SE: 97.7%; SP:96.6%; F1-Score: 96.4%
2021	Bang, et al	CNN	45	Diagnosis	AC: erythema (99.17%); papulation (93.17%); Excoriation (96.00%); lichenification (97.17%)
2022	Kapil, et al	Stratified K-fold (K = 10) cross-validation	103	Diagnosis	SE: 94%; SP:85%
2022	Jiang, et al	LR, SVM, RF	35	Prediction	Average F1-Score: 0.84
2022	Jashin, et al	lasso logistic regression, elastic net logistic regression, random forests, and gradient boosted trees	48	Treatment, prediction	Successfully predicted the influencing factors
2022	Wang, et al	CNN	49	Treatment	Successfully validated potential therapeutic drugs
2023	Maulana, et al	CNN	44	Diagnosis	AC: 89.8%; SE: 89.8%; SP: 96.6%; F1-Score: 89.85%
2023	Zvulunov, et al	Atopic App mobile health app	52	Management	POME score decreased
2024	Yunzhao Xing, et al	1D-CNN	104	Management	F1-score: 45%; precision: 44%; Recall: 46%
2024	Yizhi Dong, et al	XGBoost; genetic algorithm; logistic regression	34	Prediction	AUROC eczema: XGBoost (0.611); genetic algorithm (0.571); logistic regression (0.609). Rhinitis: XGBoost (0.602); genetic algorithm (0.572); logistic regression (0.575). Wheeze: XGBoost (0.604); genetic algorithm (0.606); logistic regression (0.620).
2024	Huang, et al	U-Net Attention models, XGBoost algorithms	42	Diagnosis	KFold value: 7; SE: 90.72%; SP: 96.76%; F1-score: 90.08%; AUROC: 0.9351
2024	-	Unsupervised cluster	46	Diagnosis	Successfully distinguished between AD patients and healthy individuals
2024	Pranvera Sulejmani	ChatGPT	51	Management	Improved quality of answers

**Table 3:** Effects of artificial intelligence for the application of food allergy.

Year	Author	Specific Artificial Intelligence	Reference	Diagnose/prediction/treatment/management	efficiency
2013	Dimitrov, et al	KNN	57	Prediction	SE: 94%; SP: 94%; F1-Score: 94%
2019	Metwally, et al	Long Short-Term Memory (LSTM) networks	56	Prediction	AUROC: 0.69; MCC: 0.40
2023	Xin-Xin Yu, et al	Quantitative structure-activity Relationship (QSAR) models	105	Prediction	RMSE: 0.2375
2024	Landau, et al	Random Forest Regression	58	Prediction	AUROC: 0.8
2019	Alag, et al	A Java-based machine learning toolkit	106	Diagnose	cg06410630 and cg06669701 are higher for food-allergic patients.
2021	Kuniyoshi, et al	LR; SVM; XGBoost	60	Diagnose; prediction	LR: AUROC (82%); SE (70%); SP (73%); AC (72%) SVM: AUROC (83%); SE (68%); SP (74%); AC (72%) XGB: AUROC (63%); SE (51%); SP (66%); AC (59%)
2023	Lucy, et al	NLP	62	Diagnose	AUROC: LR (0.84); Passive-aggressive (83.0%)
2022	Pradana-López, et al	CNN	65	Treatment; management	AC: 99.1%
2024	Liu, et al	Artificial Intelligence	66	Treatment	Screen out three potential therapeutic targets: IL-5, PTAFR and RNF19B.

**Table 4:** Effects of artificial intelligence for the application of allergic rhinitis and urticaria.

Year	Author	Specific Artificial Intelligence	Reference	Diagnose/prediction/treatment/management	efficiency
2015	Christopher, et al	CBA; SVM	70	Diagnosis	CBA: SE (94.0%); SP (94.2%); precision (92.3%); AC (94.17%) SVM: SE (93.4%); SP (93.1%); precision (90.8%); AC (93.24%)
2021	Huang, et al	Random forest model	67	Prediction	AUROC: AR (84%); AD (83%)
2022	Betul, et al	CNN	71	Diagnosis	AC: 98.3%; precision: 99%; Recall: 98%
2022	Malizia, et al	Latent class analysis (LCA)	73	Diagnosis	Two subtypes of AR have been identified.
2022	Türk, et al	Cluster analysis	82	Diagnosis	Four groups were identified.
2023	Lisha, et al	smart watch	75	Management	Patients in the intervention group showed improved medication adherence and significantly reduced nasal symptoms.
2024	Fan, et al	ChatGPT	107	Management	Correctly answered 80% of the questions
2024	Simon, et al	ChatGPT	108	Management	Likert scale score: Differences in accuracy scores.
2024	Hyeon, et al	Multi-variable spatiotemporal graph convolutional network (MST-GCN)	68	Prediction	AC: AR (88%); AD (68%); Asthma (86%) R^2^: AR (96%); AD (75%); Asthma (93%)
2024	Dai, et al	Ensemble learning algorithms	69	Diagnosis	ARF-OOBEE: AUROC (98.30%); SE (89.49%); SP (98.05%); AC (97.04%) GC Forest: AUROC (95.28%); SE (89.80%); SP (98.10%); AC (97.48%)
2024	Junhyoung, et al	Artificial intelligence (AI)-based new drug development platform	74	Treatment	Successfully screened potential therapeutic drugs for AR.
2024	Hojae, et al	Random forest model	77	Management; Prediction	SE: 83.33%; SP: 82.58%; AC: 82.59%; AUROC: 89.87%
2024	Taig Mac Carthy, et al	CNN	79	Diagnosis	BAC: 0.72
2024	Lee1, et al	Deep learning	80	Diagnosis	Wheal: SE (56.21%); SP (99.95%); AC (99.85%) Erythema: SE (57.87%); SP (97.97%); AC (96.60%)

## Abbreviations

NLP: natural language processing
BiLSTM: bidirectional long-short-term memory network
TDNN: time delay neural network
PPA: Positive Percent Agreement
NPA: Negative Percent Agreement
RCNN: recurrent-convolutional neural network
JDINAC: joint density-based nonparametric differential interaction network analysis and classification
ANN: artificial neural network
SVN: support vector machine
KNN: k-nearest neighbour
SVR: support vector regression
RF: random forest
NBC: naive Bayesian classifier
ABN: adaptive Bayesian network
SE: sensitivity
SP: specificity
AC: accuracy
PPV: positive predictive value
NPV: negative predictive value
ANSN: average NPV-Specificity area
AUROC: area under the receiver operating characteristic curve
NNA: neural network analysis
MLA: multiple logistic regression analysis
CNN: convolutional neural networks
LR: logistic regression
MCC: Matthews correlation coefficient
RMSE: root mean squared error
CBA: associative classifier
BAC: balanced accuracy
R^2^: coefficient of determination
COPD: chronic obstructive pulmonary disease
